# PANoptosis In Microglia and Neurons Following Traumatic CNS Injury: A Systematic Review of Pathological Features and Therapeutic Implications

**DOI:** 10.1002/cns.71208

**Published:** 2026-10-13

**Authors:** Zhusheng Feng, Yingnan Fan, Tianshu Du, Peipei Huang, Yuqin Ye, Junjie Li

**Affiliations:** ^1^ Department of Emergency Xijing Hospital, The Fourth Military Medical University Xi'an Shaanxi China; ^2^ Department of Healthcare Management Tangdu Hospital, The Fourth Military Medical University Xi'an Shaanxi China; ^3^ Department of Nursing The Fourth Military Medical University Xi'an Shaanxi China; ^4^ Department of Orthopaedics Xijing Hospital, The Fourth Military Medical University Xi'an Shaanxi China; ^5^ Department of Neurosurgery Xijing Hospital, The Fourth Military Medical University Xi'an Shaanxi China

**Keywords:** intervention, mechanism, microglia, neuron, PANoptosis, PANoptosome, spinal cord injury, traumatic brain injury

## Abstract

**Background:**

Traumatic brain injury (TBI) and spinal cord injury (SCI) induce PANoptosis, a pro‐inflammatory programmed cell death modality orchestrated by the PANoptosome. This modality integrates pyroptosis, apoptosis, and necroptosis molecular machinery and contrasts with canonical single‐pathway cell death.

**Methods:**

This systematic review (PROSPERO CRD420261383435) followed PRISMA and searched PubMed, Web of Science, and Cochrane Library. The in vivo studies confirming PANoptosis at the protein level were included; quality was assessed using CRIME‐Q, and shared genes/protein interactions were analyzed via GeneCards, STRING, and Cytoscape.

**Results:**

Ten studies (five TBI and five SCI) were included. PANoptosis occurs in microglia and neurons, with significant crosstalk in secondary injury. Regulatory mechanisms included transcriptional regulation, inflammatory signaling, ubiquitination, energy metabolism, proteolysis, and mitophagy. Interventions comprised specific inhibitors, Chinese medicine formula granules, small molecules, siRNAs, drugs, metabolites, plasmids, and metal ions. Key sensors included ZBP1 and NLRP12, with ASC as the main adaptor; executioners included CASP1, GSDMD, CASP3/8, RIPK1/3, MLKL, and activated forms. Ischemia was the most significant disease‐gene network factor.

**Conclusion:**

The primary injury activates microglia and neurons, inducing PANoptosis via multiple pathways; targeting PANoptosome assembly may preserve immuno‐inflammatory balance and limit secondary damage. Future research should prioritize comprehensive PANoptosome detection, standardized animal models, CNS cell interactions, novel drug delivery, and ischemia–reperfusion injury.

## Graphical abstract

Following traumatic CNS injury, PANoptosis is activated in microglia and neurons, with microglia–neuron crosstalk serving as a key feature of this process. The PANoptosome sensors ZBP1 and NLRP12, together with the adaptor ASC, activate downstream executioner proteins to drive PANoptosis. PANoptosis is regulated through transcriptional, inflammatory, ubiquitin‐dependent, metabolic, proteolytic, and mitophagic pathways. Therapeutic strategies targeting PANoptosis attenuate secondary injury and preserve immune and inflammatory homeostasis.

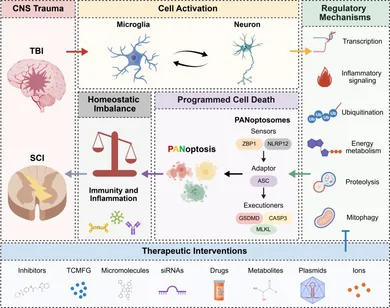

## 1 Introduction

Traumatic central nervous system (CNS) injury, encompassing traumatic brain injury (TBI) and spinal cord injury (SCI), is characterized by structural damage and functional impairment resulting from external mechanical forces [1]. The pathological evolution of TBI and SCI is not a single event. Instead, it is a complex spatiotemporal cascade initiated by a primary mechanical insult and perpetuated by secondary injury mechanisms [2, 3]. Collectively, these conditions are among the leading causes of disability and mortality worldwide and constitute a major public health burden [4]. The pathophysiology of TBI and SCI is multifaceted and includes neuroinflammatory responses [5], immune microenvironment remodeling [6], integrated regulation of cell death pathways [7, 8], oxidative stress [9], accumulation of metabolic toxins [10], glial scarring and fibrotic microenvironment [11, 12], proteinopathological changes [13], ion channel dysfunction [14], and immune crosstalk between the brain and peripheral organs. These interrelated processes are inextricably linked and collectively play critical roles in the onset and progression of TBI and SCI [15].

Microglia and neurons are essential for maintaining CNS homeostasis and facilitating damage repair [16, 17]. As the principal resident immune cells of the CNS, microglia mediate immune surveillance under homeostatic conditions [18]. Following TBI and SCI, microglia undergo rapid proliferation and exhibit altered gene and protein expression. Through cytokine release, they recruit peripheral immune cells and participate in both inflammatory responses and neural structural repair [19]. Microglia play a significant dual role in injury. Early activation exerts a protective effect by facilitating the clearance of cellular debris and promoting neuroplastic repair [20]. However, sustained stimulation leads to microglial overactivation. This leads to the release of pro‐inflammatory and cytotoxic mediators that drive neuroinflammation, oxidative stress, and mitochondrial dysfunction [21]. These effects compromise neuronal survival and synaptic plasticity, disrupt neural circuit integrity, exacerbate secondary injury, and impede functional recovery [22]. The inherently limited capacity for axonal regeneration in adult CNS neurons renders TBI and SCI particularly prone to permanent neurological disabilities [23]. TBI can damage long‐projecting axons and white matter fiber tracts. This damage impairs information transmission and causes neural circuit dysfunction [24]. Following TBI and SCI, neurons may undergo various forms of programmed cell death (PCD), including mitophagy, pyroptosis, ferroptosis, and necroptosis [25].

PANoptosis is a newly recognized multimodal form of inflammatory PCD. It integrates the molecular machinery of pyroptosis, apoptosis, and necroptosis [26, 27]. Unlike its individual constituent pathways, PANoptosis operates as an independent modality that cannot be prevented by inhibiting any single PCD pathway [28]. The PANoptosome is a central multiprotein complex that orchestrates PANoptosis. It requires sequential assembly and cascading activation to induce cell death [29]. This complex contains innate immune sensors, adaptor proteins, and catalytic executioners. The sensors recognize pathogen‐associated molecular patterns (PAMPs) or damage‐associated molecular patterns (DAMPs). The concurrent activation of all three PCD pathways by distinct executioner proteins results in cell lysis and the release of pro‐inflammatory cytokines, DAMPs, and alarmins [30, 31]. PANoptosomes are classified into Z‐DNA binding protein 1 (ZBP1)‐, absent in melanoma 2 (AIM2)‐, receptor‐interacting protein kinase 1 (RIPK1)‐, NOD‐like receptors family pyrin domain containing 12 (NLRP12)‐, NLR family CARD domain containing 5 (NLRC5)‐, and NLRP3‐mediated subtypes according to their sensor composition [32].

Our previous investigation of the interplay between PANoptosis and disrupted mitochondrial homeostasis indicated that therapeutic modulation of this mechanism holds considerable promise for improving the prognosis following TBI and SCI [33]. Based on these findings, the present systematic review examines cell type‐specific PANoptosis induced by TBI and SCI. It also addresses PANoptosome‐driven molecular pathophysiology and therapeutic strategies, with the aim of providing a comprehensive evidence‐based synthesis to inform future research.

## 2 Methods and Materials

### 2.1 Search Strategy

This systematic review was prospectively registered with the international prospective register of systematic reviews (PROSPERO) (registration number: CRD420261383435) and conducted in accordance with the preferred reporting items for systematic reviews and meta‐analyses (PRISMA) guidelines (Table S1). Systematic searches were performed across PubMed, Web of Science, and the Cochrane Library. A comprehensive search strategy was implemented using both Medical Subject Headings (MeSH) terms and free‐text keywords applied to all fields. The search terms included: “Brain Injuries, Traumatic,” “Traumatic Brain Injury,” “Brain Trauma,” “Spinal Cord Injuries,” “Spinal Cord Injury,” “Spinal Cord Trauma,” “PANoptosis,” “PANoptosome,” and “PANoptotic.” The search was restricted to records published from database inception to August 12, 2026 (Table S2–S4).

### 2.2 Inclusion and Exclusion Criteria

The inclusion criteria were as follows: (i) original research articles; (ii) studies focusing on traumatic CNS injury, including TBI and SCI; (iii) studies investigating PANoptosis; (iv) studies incorporating protein‐level experimental validation; and (v) studies that included PANoptosome detection. The exclusion criteria were as follows: (i) non‐original research articles (e.g., reviews, editorials, commentaries); (ii) studies not involving TBI or SCI; (iii) studies not addressing PANoptosis; (iv) studies relying exclusively on genomic, transcriptomic, proteomic, or metabolomic analyses without complementary experimental validation; (v) studies lacking protein‐level detection; (vi) studies lacking PANoptosome detection; (vii) studies without an in vivo experimental component; and (viii) studies lacking investigation of molecular mechanisms or intervention strategies.

### 2.3 Data Collection, Risk of Bias, and Assessment of Study Quality

Two reviewers (Z.F. and Y.F.) independently performed data extraction, risk of bias assessments, and study quality appraisals. Discrepancies were resolved through consultation with a third reviewer. The extracted data included disease type, experimental model, cell type, experimental methods, PANoptosome composition, regulatory molecules, signaling pathways, and intervention strategies. The risk of bias and study quality in animal experiments were assessed using the critical appraisal of methodological (technical) quality, quality of reporting, and risk of bias in animal research (CRIME‐Q) tool, with responses categorized as yes, partly/unclear, no, or not applicable (NA) [34].

### 2.4 Data Analysis and Graph Generation

The literature screening flowchart and study quality assessment diagram were generated using draw.io (https://app.diagrams.net/). GeneCards [35] (version 6.1; https://www.genecards.org/) was used to identify genes associated with PANoptosis in TBI and SCI. A relevance score threshold of ≥ 5 was applied, and the analysis was restricted to protein‐coding genes. Jvenn [36] was used to generate intersection diagrams of genes common to PANoptosis in TBI and SCI. Search tool for the retrieval of interacting genes/proteins (STRING) [37] (version 12.0; https://cn.string‐db.org/) was used to generate enrichment diagrams for Kyoto Encyclopedia of Genes and Genomes (KEGG), Reactome, Gene Ontology (GO), and Disease‐gene associations among the shared genes. The organism parameter was set to *Homo sapiens* . Cytoscape [38] (version 3.10.4; https://cytoscape.org/) was used to analyze gene interaction networks. BioGDP [39] was used to construct mechanistic schematic diagrams.

## 3 Results

A total of 62 records were retrieved from the three databases (30 from PubMed, 32 from Web of Science, and 0 from the Cochrane Library). Ultimately, 10 studies [40, 41, 42, 43, 44, 45, 46, 47, 48, 49] met all the inclusion criteria and were included in the qualitative synthesis (Figure S1).

### 3.1 Risk of Bias and Quality Assessment

The risk of bias and quality assessment results for the 10 included animal studies [40, 41, 42, 43, 44, 45, 46, 47, 48, 49] are presented in Figure S2.

### 3.2 Characteristics of the Included Experiments

Ten studies were published between 2024 and 2026, comprising five TBI [41, 42, 45, 46, 47] and five SCI studies [40, 43, 44, 48, 49]. All experiments were conducted in rodents: eight studies used C57BL/6 mice [41, 42, 44, 45, 46, 47, 48, 49], one used Sprague–Dawley (SD) rats [40], and one used Wistar rats [43]. All TBI models were induced using controlled cortical impact (CCI), whereas SCI models were induced using either contusion or CCI. TBI lesions were predominantly localized to the left or right cerebral cortex, and SCI lesions were primarily centered at the thoracic level (predominantly T10). For TBI, the CCI parameters were as follows: impact velocity, 3.0–6.0 m/s; depth, 0.6–3.0 mm; and duration, 0.05–0.1 s. One study employed blast injury using air pressure (100/200 kPa) as a model parameter. For SCI, the CCI parameters were as follows: velocity, 0.5 m/s; depth, 1.0 mm; and duration, 1 s. Contusion models involved a falling weight (5.0–10.0 g dropped from 25 to 30 mm), and one study specified an impact force of 50.0 kilodyne (kdyn). Neurons and microglia were identified using neuronal nuclei (NeuN) and ionized calcium‐binding adapter molecule 1 (Iba1) immunolabeling in the majority of studies. The temporal window for PANoptosis detection ranged from 1 to 21 days post‐TBI, whereas post‐SCI assessments were typically performed on Days 3 and 7 (Table S5).

### 3.3 Detection and Composition of PANoptosomes

All studies employed western blot (WB) as the primary analytical method, supplemented by immunofluorescence (IF), immunohistochemistry (IHC), flow cytometry (FCM), and enzyme‐linked immunosorbent assay (ELISA), among other techniques. WB was used for protein quantification, IF/IHC for spatial localization, and FCM/ELISA for cellular‐level analysis. Protein expression and post‐translational modifications of PANoptosome‐related molecules were evaluated using these complementary approaches. The PANoptosome triggers identified in the included studies comprised DAMPs induced by TBI and SCI, together with specific mediators, including 3‐methylbutanoic acid (3‐MA) [41], heme [42], tumor necrosis factor‐α (TNF‐α) [42], myelin debris [44], and reactive oxygen species (ROS) [47], as well as the exogenous compound evodiamine (EVO) [46]. All included studies examined the executioners of PANoptosis; however, two studies did not assess the sensors and adaptors of the PANoptosome. The principal sensors were ZBP1 [40, 42, 46, 48] and NLRP12 [44]. The predominant adaptor protein was apoptosis‐associated speck‐like protein containing a CARD (ASC) [41, 43, 45, 46]. Executioner proteins included caspase‐1 (CASP1), gasdermin D (GSDMD), CASP3, CASP8, RIPK1/3, and mixed lineage kinase domain‐like pseudokinase (MLKL), along with their activated forms (Table 1 and Figure 1).

**TABLE 1:** Experimental detection for PANoptosomes.

Test method	Trigger	Sensor	Adaptor	Executioner	References
WB, IF, ELISA	DAMPs	ZBP1	NR	GSDMD, GSDMD‐N, C‐CASP3, RIPK, p‐RIPK, MLKL, p‐MLKL	Xu et al. [40]
WB, IF, TUNEL	3‐MA	NR	ASC	GSDMD‐N, RIPK3, p‐RIPK3, CASP8, C‐CASP8, MLKL, p‐MLKL	Xie et al. [41]
WB, IF, FCM	Heme, TNF‐α	ZBP1	NR	CASP1, GSDMD, GSDME, CASP8, CASP3, CASP7, MLKL, p‐MLKL, RIPK3, p‐RIPK3, C‐CASP3	Shuang et al. [42]
WB, IF, FCM, ELISA, TUNEL	DAMPs	NR	ASC	GSDMD, CASP1, GSDMD‐N, C‐CASP1, CASP3, C‐CASP3, RIPK1, RIPK3, MLKL	Liu et al. [43]
WB, IF Co‐IP	Myelin debris	NLRP12	RIPK3, CASP8	CASP3, C‐CASP3, GSDMD, GSDMD‐N, p‐MLKL, MLKL, C‐CASP1	Jiang et al. [44]
WB, IF	DAMPs	NS	ASC	CASP1, GSDMD, CASP3, RIPK1, RIPK3	Chen et al. [45]
WB, IF, FCM	EVO	ZBP1	AIM2, ASC, Pyrin	CASP1, C‐CASP1, GSDMD, GSDMD‐N, CASP8, CASP3, C‐CASP3, RIPK1, RIPK3, MLKL	Zheng et al. [46]
WB, TUNEL, PI, EthD‐III	ROS	NR	NR	CASP8, C‐CASP8, CASP3, C‐CASP3, p‐RIPK1, RIPK1, p‐RIPK3, RIPK3, p‐MLKL, MLKL, CASP1, C‐CASP1, GSDMD, GSDMD‐N	Xu et al. [47]
WB, IHC	DAMPs	ZBP1	NR	CASP1, C‐CASP1, GSDMD‐N, CASP3, C‐CASP3, RIPK3, p‐RIPK3, MLKL, p‐MLKL	Lou et al. [48]
WB, IF	DAMPs	NR	NR	CASP1, GSDMD, CASP3, C‐CASP, RIPK1, RIPK3, MLKL	Bai et al. [49]

Abbreviations: 3‐MA, 3‐methylbutanoic acid; AIM2, absent in melanoma 2; ASC, apoptosis‐associated speck‐like protein containing a CARD; CASP, caspase; C‐CASP, cleaved‐caspase; Co‐IP, immunoprecipitation; DAMPs, damage‐associated molecular patterns; ELISA, enzyme‐linked immunosorbent assay; EthD‐III, ethidium homodimer III; EVO, evodiamine; FCM, flow cytometry; GSDMD, gasdermin D; GSDME, gasdermin E; GSDMD‐N, N‐terminal domain of gasdermin D; IF, immunofluorescence; IHC, immunohistochemistry; MLKL, mixed lineage kinase domain‐like pseudokinase; NLRP12, NOD‐like receptors family pyrin domain containing 12; NR, not reported; NS, not specified; PI, propidium iodide; p‐MLKL, phosphorylation of mixed lineage kinase domain‐like pseudokinase; p‐RIPK, phosphorylation of receptor‐interacting protein kinase; RIPK, receptor‐interacting protein kinase; ROS, reactive oxygen species; TNF‐α, tumor necrosis factor‐α; TUNEL, TdT‐mediated dUTP nick‐end labeling; WB, western blot; ZBP1, Z‐DNA binding protein 1.

**FIGURE 1:**
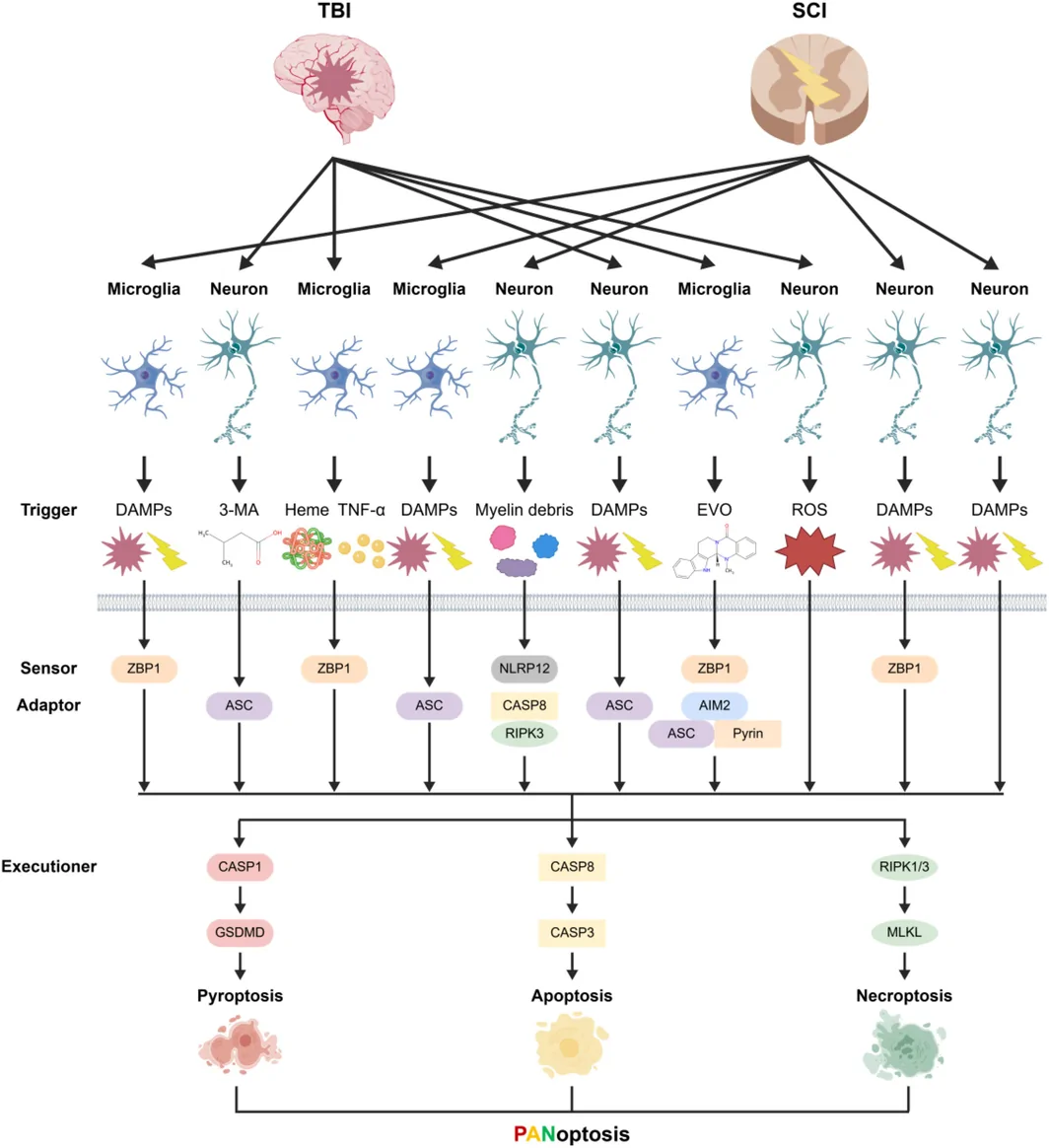
Schematic representation of the molecular mechanisms in PANoptosomes. 3‐MA, 3‐methylbutanoic acid; AIM2, absent in melanoma 2; ASC, apoptosis‐associated speck‐like protein containing a CARD; CASP, caspase; DAMPs, damage‐associated molecular patterns; EVO, evodiamine; GSDMD, gasdermin D; MLKL, mixed lineage kinase domain‐like pseudokinase; NLRP 12, NOD‐like receptors family pyrin domain containing 12; RIPK, receptor‐interacting protein kinase; ROS, reactive oxygen species; TNF‐α, tumor necrosis factor‐α; ZBP1, Z‐DNA binding protein 1.

### 3.4 Molecular Mechanisms of PANoptosis in TBI and SCI

The included studies identified regulatory mechanisms spanning transcriptional control (interferon regulatory factor 1 [IRF1] and Y‐box binding protein 1 [YBX1]) [40, 48], inflammation (TNF‐α) [41], ubiquitination (ubiquitin‐specific protease 4 [USP4], proteasome 26S subunit, non‐ATPase 14 [PSMD14], and tripartite motif containing 56 [TRIM56]) [42, 47, 48], energy metabolism (adenosine monophosphate‐activated protein kinase [AMPK]) [43], proteolysis (cathepsin D [CTSD]) [45], and mitophagy (PTEN‐induced kinase 1 [PINK1] and Bcl‐2‐associated X protein [BAX]) [47, 49]. ZBP1 was identified as a consistently upregulated PANoptosome sensor across four studies [40, 42, 46, 48], with its upregulation documented in both neuronal and microglial populations following TBI and SCI. NLRP12 is directly activated by myelin debris [44] (Table 2 and Figure 2).

**TABLE 2:** Molecular mechanisms of PANoptosis in TBI and SCI.

Disease	PANoptotic Cell	Regulated molecule	Molecular alteration	Effect on PANoptosis	References
SCI	Microglia	IRF1, ZBP1	Upregulated	Promotes	Xu et al. [40]
TBI	Neuron	TNF‐α	Upregulated	Promotes	Xie et al. [41]
TBI	Microglia	AKT, USP4, ZBP1	Upregulated	Promotes	Shuang et al. [42]
SCI	Microglia	AMPK	Downregulated	Promotes	Liu et al. [43]
SCI	Neuron	NLRP12	Upregulated	Promotes	Jiang et al. [44]
TBI	Neuron	Snapin, m‐CTSD	Upregulated	Promotes	Chen et al. [45]
		H_2_S	Downregulated		
TBI	Microglia	ZBP1	Upregulated	Promotes	Zheng et al. [46]
TBI	Neuron	PSMD14, PKM, PINK1	Upregulated	Inhibits	Xu et al. [47]
		ROS	Downregulated		
SCI	Neuron	TRIM56	Downregulated	Promotes	Lou et al. [48]
		YBX1, ZBP1	Upregulated		
SCI	Neuron	LGALS3, BAX	Upregulated	Promotes	Bai et al. [49]

Abbreviations: AKT, protein kinase B; AMPK, adenosine monophosphate‐activated protein kinase; BATF, basic leucine zipper ATF‐like transcription factor; BAX, Bcl‐2‐associated X protein; CTSD, cathepsin D; H_2_S, hydrogen sulfide; IRF1, interferon regulatory factor 1; LGALS3, lectin, galactoside‐binding, soluble, 3; m‐CTSD, mature CTSD; NLRP12, NLR family pyrin domain containing 12; PINK1, PTEN induced kinase 1; PKM, pyruvate kinase M; PSMD14, proteasome 26S subunit, non‐ATPase 14; ROS, reactive oxygen species; SCI, spinal cord injury; TBI, traumatic brain injury; TRIM56, tripartite motif containing 56; TNF‐α, tumor necrosis factor‐α; USP4, ubiquitin‐specific protease 4; YBX1, Y‐box binding protein 1; ZBP1, Z‐DNA binding protein 1.

**FIGURE 2:**
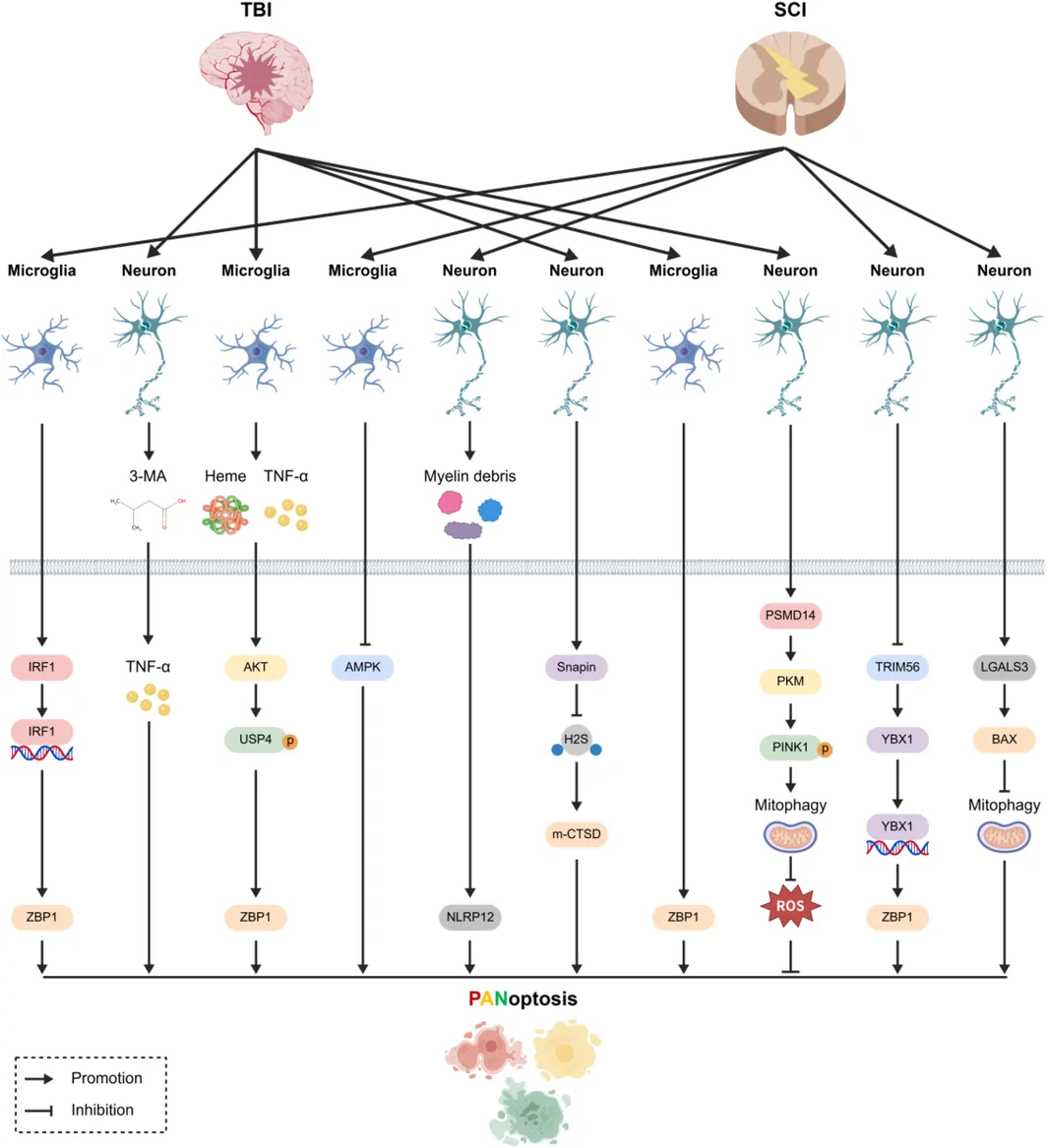
Regulatory mechanisms of PANoptosis in TBI and SCI. 3‐MA, 3‐methylbutanoic acid; AKT, protein kinase B; AMPK, adenosine monophosphate‐activated protein kinase; BATF, basic leucine zipper ATF‐like transcription factor; BAX, Bcl‐2‐associated X protein; CTSD, cathepsin D; H_2_S, hydrogen sulfide; IRF1, interferon regulatory factor 1; LGALS3, lectin, galactoside‐binding, soluble, 3; m‐CTSD, mature CTSD; NLRP12, NLR family pyrin domain containing 12; PINK1, PTEN‐induced kinase 1; PKM, Pyruvate kinase M; PSMD14, proteasome 26S subunit, non‐ATPase 14; ROS, reactive oxygen species; SCI, spinal cord injury; TBI, traumatic brain injury; TNF‐α, tumor necrosis factor‐α; TRIM56, tripartite motif containing 56; USP4, ubiquitin‐specific protease 4; YBX1, Y‐box binding protein 1; ZBP1, Z‐DNA binding protein 1.

### 3.5 Intervention Strategies for PANoptosis in TBI and SCI

Intervention strategies encompassed specific inhibitors (interferon regulatory factor 1 inhibitor 1 [IRF1‐IN‐1] and 3,6′‐dithiothalidomide [3,6′‐DT], and pepstatin A) [40, 41, 45], traditional Chinese medicine formula granules (TCMFG) (refined Changqin No. 1 [R‐Cq1]) [41], small‐molecule compounds (vialinin A, endogenous H_2_S, and EVO) [42, 45, 46], small interfering RNA (siRNA) (small interfering RNA against signal regulatory protein alpha [siSIRPα]) [44], drugs (metformin) [43], metabolites (lactic acid) [47], plasmids (adeno‐associated virus‐short hairpin RNA [AAV‐shRNA] and TRIM56‐Flag) [45, 48], and ions (zinc) [49]. Notably, metabolism‐targeted interventions—including metformin (AMPK) [43], lactate (PSMD14) [47], H_2_S (mature‐CTSD) [45], and zinc ions (LGALS3) [49]‐have demonstrated promising therapeutic potential (Table 3 and Figure 3).

**TABLE 3:** Therapeutic interventions for PANoptosis in TBI and SCI.

Intervention	Intervention mechanism	Effect on PANoptosis	References
IRF1‐IN‐1 (IRF1 inhibitor)	RF1 nuclear translocation is associated with increased expression of ZBP1 in microglia, which can be inhibited by IRF1‐IN‐1.	Inhibits	Xu et al. [40]
R‐Cq1, 3,6′‐DT (TNF inhibitor)	R‐Cq1 suppresses Anaerotruncus‐associated 3‐MA to hinder TNF‐α upregulation, thereby alleviating neuronal PANoptosis and TBI. When TNF‐α is inhibited, the pro‐PANoptotic effect of 3‐MA is attenuated.	Inhibits	Xie et al. [41]
Vialinin A	Pharmacological inhibition of USP4 using vialiinin A leads to ZBP1 degradation, reduced microglial PANoptosis, and amelioration of TBI‐related functional deficits.	Inhibits	Shuang et al. [42]
Metformin	Metformin alleviates microglial PANoptosis in an AMPK‐dependent manner, promoting tissue repair and functional recovery after SCI.	Inhibits	Liu et al. [43]
siSIRPα	Inhibition of Sirpα expression enhances myelin debris clearance by microglia through activation of the SYK/PI3K/AKT pathway and suppression of NLRP12‐mediated PANoptosis of neurons, effectively reducing neuronal death and promoting neurological recovery after SCI.	Inhibits	Jiang et al. [44]
AAV‐shSnapin, Endogenous H_2_S, pepstatin A (CTSD inhibitor)	AAV‐shSnapin modulates H_2_S homeostasis after mTBI, and molecular docking combined with coimmunoprecipitation confirmed a direct interaction between Snapin and H_2_S‐synthesizing enzymes. Pepstatin A suppresses CTSD activity, primarily by inhibiting its maturation. In parallel, exogenous H_2_S reduced m‐CTSD expression after TBI.	Inhibits	Chen et al. [45]
EVO	EVO selectively induces PANoptosis in microglia, thereby disrupting pro‐inflammatory activity.	Promotes	Zheng et al. [46]
Lactic acid	Lactate treatment alleviates neuron PANoptosis by increasing PSMD14 expression.	Inhibits	Xu et al. [47]
TRIM56‐Flag, AAV‐shYBX1, AAV‐shZBP1	YBX1 promotes ZBP1 expression by stabilizing ZBP1 mRNA, thereby aggravating ZBP1‐mediated PANoptosis. Furthermore, the E3 ubiquitin ligase TRIM56 has been identified as an endogenous inhibitor of YBX1.	Inhibits	Lou et al. [48]
Zinc ions	Zinc ions play a key role in mitigating mitochondrial quality control dysfunction triggered by oxidative stress, and this is achieved by targeting the LGALS3‐BAX pathway.	Inhibits	Bai et al. [49]

Abbreviations: H3,6′‐DT, 3,6′‐dithiothalidomide; 3‐MA, 3‐methylbutanoic acid; AAV, adeno‐associated virus; AIM2, absent in melanoma 2; AKT, protein kinase B; AMPK, adenosine monophosphate‐activated protein kinase; BAX, Bcl‐2‐associated X protein; CTSD, cathepsin D; EVO, evodiamine; H_2_S, hydrogen sulfide; IRF1‐IN‐1, interferon regulatory factor 1 inhibitor 1; LGALS3, galectin‐3; m‐CTSD, mature CTSD; NLRP12, NOD‐like receptors family pyrin domain containing 12; PI3K, phosphatidylinositol 3‐kinase; PSMD14, proteasome 26S subunit, non‐ATPase 14; R‐Cq1, Refined Changqin No. 1; SCI, spinal cord injury; shRNA, short hairpin RNA; siRNA, small interfering RNA; SIRPα, signal regulatory protein alpha; SKY, spleen tyrosine kinase; TBI, traumatic brain injury; TRIM56, tripartite motif containing 56; TNF‐α, tumor necrosis factor‐α; USP4, ubiquitin‐specific protease 4; YBX1, Y‐box binding protein 1; ZBP1, Z‐DNA binding protein 1.

**FIGURE 3:**
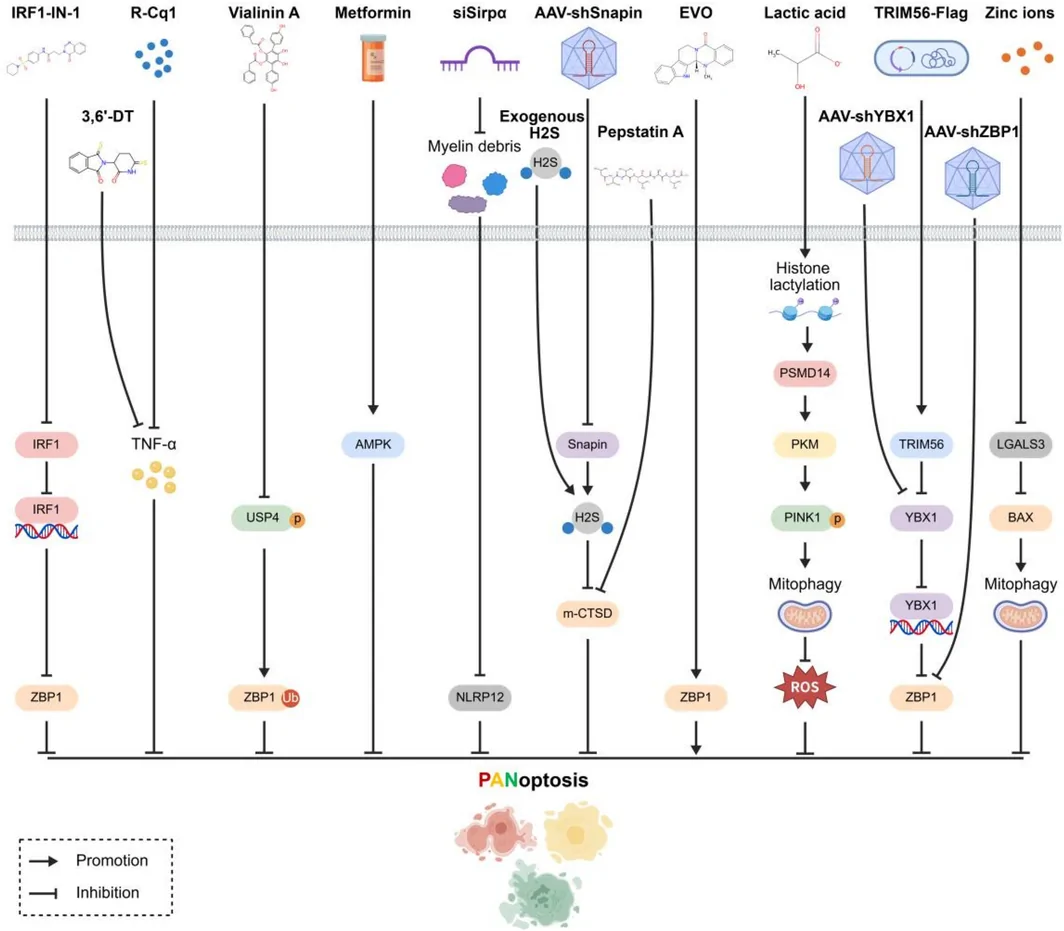
Intervention mechanisms of PANoptosis in TBI and SCI. 3,6′‐DT, 3,6′‐dithiothalidomide; 3‐MA, 3‐methylbutanoic acid; AAV, adeno‐associated virus; AIM2, absent in melanoma 2; AKT, protein kinase B; AMPK, adenosine monophosphate‐activated protein kinase; BAX, Bcl‐2‐associated X protein; CTSD, cathepsin D; EVO, evodiamine; H_2_S, hydrogen sulfide; IRF1‐IN‐1, interferon regulatory factor 1 inhibitor 1; LGALS3, galectin‐3; m‐CTSD, mature CTSD; NLRP12, NOD‐like receptors family pyrin domain containing 12; PI3K, phosphatidylinositol 3‐kinase; PINK1, PTEN‐induced kinase 1; PKM, pyruvate kinase M; PSMD14, proteasome 26S subunit, non‐ATPase 14; R‐Cq1, Refined Changqin No. 1; SCI, spinal cord injury; shRNA, short hairpin RNA; siRNA, Small interfering RNA; SIRPα, signal regulatory protein alpha; SKY, spleen tyrosine kinase; TBI, traumatic brain injury; TRIM56, tripartite motif containing 56; TNF‐α, tumor necrosis factor‐α; USP4, ubiquitin‐specific protease 4; YBX1, Y‐box binding protein 1; ZBP1, Z‐DNA binding protein 1.

### 3.6 Microglia–Neuron Crosstalk in PANoptosis

Inhibition of SIRPα expression in microglia enhances myelin debris clearance through activation of the spleen tyrosine kinase (SYK)/phosphatidylinositol 3‐kinase (PI3K)/protein kinase B (AKT) pathway. This inhibition also suppresses NLRP12‐mediated PANoptosis in neurons. Consequently, neuronal death is reduced and neurological recovery is promoted [44]. DAMPs‐activated microglia exacerbate neuronal injury through multiple mechanisms. They phagocytose neuronal structures, including axons, dendrites, and synapses. They also secrete pro‐inflammatory cytokines and disrupt mitochondrial integrity and energy metabolism. These combined effects ultimately lead to neuronal dysfunction or death. EVO counteracts these deleterious effects by promoting PANoptosome assembly through the upregulation of PANoptosis‐associated proteins in microglia [46] (Figure S3).

### 3.7 Supplementary Bioinformatics Analysis

Based on the direct evidence from the systematic review described above, we performed bioinformatics analysis to identify the genes commonly involved in PANoptosis in TBI and SCI. Eighteen shared genes (*CASP3, HIF1A, HMGB1, IL1B, MAPK3, MTOR, PTGS2, TNF, NFKB1, HMOX1, NFE2L2, SIRT1, TLR4, CASP1, PARP1, MMP2, NLRP3*, and *MAPK1*) were interconnected within a unified network. Disease‐gene association enrichment analysis identified ischemia as the most significantly associated disease (Figure S4).

## 4 Discussion

The 10 included studies were peer‐reviewed, compliant with animal welfare regulations, and disclosed any potential conflicts of interest. Model establishment and technical execution were robust, outcome data were complete, selective reporting was absent, and the risk of publication bias was low in this study. However, methodological limitations were evident in terms of randomization, allocation concealment, blinded outcome assessment, and sample size calculation.

The included studies showed substantial heterogeneity in model parameters, disease severity, and time course. However, owing to the limited number of studies included, no formal subgroup analysis was performed. The CCI model was predominantly used for TBI, whereas the contusion model was primarily employed for SCI. Even within the same model, parameters such as impact velocity, depth, duration, and pressure vary. Differences in model parameters directly influence injury severity. They affect both primary injury and secondary inflammatory responses and consequently modulate the PANoptosis‐mediated pathological process.

Among the included studies, Zheng et al. [46] demonstrated that EVO induces PANoptosis in microglia. This process exerts a protective effect through the clearance of inflammatory cells, thereby offering a novel therapeutic strategy for severe TBI. Notably, the spatial patterns of cerebral atrophy differ markedly according to TBI severity: mild TBI is characterized predominantly by gray matter atrophy, whereas moderate‐to‐severe TBI involves both subcortical gray and white matter degeneration [50]. TBI severity is independently predictive of 7‐day mortality, with severe TBI conferring a significantly higher risk of death than mild TBI [51]. Furthermore, moderate‐to‐severe and penetrating TBI are associated with an elevated risk of subsequent brain cancer, an association not observed following mild TBI [52]. The incidence of post‐traumatic epilepsy also increases with injury severity, occurring most frequently in severe cases [53]. Similarly, SCI severity profoundly modulates the extent of immune, endocrine, and multisystem dysfunction [54].

In the included studies, the temporal window for PANoptosis detection in TBI models ranged from Days 1 to 21, encompassing the acute and chronic phases. In contrast, SCI studies were largely restricted to the early post‐injury period (Days 3 and 7). The role of PANoptosis in the pathological process may differ across disease stages.

Xu et al. [47] demonstrated that upregulation of PSMD14/pyruvate kinase M (PKM)/PINK1 and concomitant downregulation of ROS inhibit PANoptosis. This represents an endogenous protective mechanism. Targeted intervention against PSMD14 yielded favorable outcomes at 21 days post‐CCI. Following TBI, both immediate and delayed pathophysiological alterations occur. The delayed phase is characterized by neuroinflammation, oxidative damage, apoptosis, and glutamate excitotoxicity [55]. Throughout the acute, subacute, and chronic phases, mitochondria‐centered oxidative stress dysregulation, energy metabolic disruption, and calcium homeostasis impairment serve as central drivers of secondary injury progression [56]. SCI pathophysiology is similarly time‐dependent, with chronic‐phase characteristics varying according to injury severity and completeness [57]. Following murine SCI, immune infiltration peaks at 2 weeks and 2 months. T‐cell and macrophage populations remain elevated at 6 months. Although acute inflammation impairs sensory function, inflammation persisting to 6 months appears to facilitate recovery and correlates with increased splenic mass [58].

PANoptosome triggers comprised DAMPs, 3‐MA, heme, TNF‐α, myelin debris, ROS, and EVO. In TBI and SCI, DAMPs release and accumulation constitute central mechanisms that mediate secondary injury, drive neuroinflammation, and influence clinical prognosis [59, 60]. DAMPs release is intimately linked to multiple regulated cell death pathways, including necroptosis, pyroptosis, and ferroptosis [61, 62]. For example, thrombin activated following SCI induces astrocytic production of macrophage migration inhibitory factor via protease‐activated receptor 1 signaling [63]. Furthermore, the blood–brain barrier (BBB) and glymphatic dysfunction promote the aberrant accumulation of DAMPs within the CNS. This accumulation represents a primary driver of sustained microenvironmental disruption [64].

The principal sensors were ZBP1 and NLRP12, the predominant adaptor was ASC, and the executioner proteins included CASP1, GSDMD, CASP3/8, RIPK1/3, and MLKL, along with their activated forms. PANoptosome‐mediated PANoptosis plays a central role in neuroinflammation and secondary injury following TBI and SCI [65]. Our previous study demonstrated that PANoptosome activation is closely associated with disrupted mitochondrial homeostasis. This disruption manifests as impaired mitochondrial biogenesis, aberrant mitochondrial dynamics, and dysfunctional mitophagy [33]. Under pathological conditions, PANoptosis may exert dual effects: it can exacerbate tissue damage and compromise prognosis while simultaneously exerting protective effects through the clearance of damaged cells [26].

The regulation of PANoptosis in TBI and SCI encompasses multiple signaling pathways, including transcriptional control (IRF1 and YBX1), inflammatory signaling (TNF‐α), ubiquitin‐mediated proteostasis (USP4, PSMD14, and TRIM56), metabolic regulation (AMPK), proteolytic processing (CTSD), and mitochondrial quality control through mitophagy (PINK1 and BAX). ZBP1 was consistently upregulated in both neuronal and microglial populations across the four studies, whereas NLRP12 was directly activated by myelin debris. Multiple transcription factors are directly involved in regulating injury‐associated gene expression [66]. Following TBI, the upstream transcriptional regulator purine‐rich box 1 mediates dynamic enhancer remodeling and transcriptional activation in circulating monocytes [67]. Post‐injury cytokine release recruits resident and infiltrating immune cells, thereby initiating a dynamic neuroinflammatory cascade [68]. Cytokines and chemokines exhibit temporal functional pleiotropy across the acute, subacute, and chronic phases. Precise modulation of their activity is essential for disrupting the self‐perpetuating inflammatory cycle [69, 70]. Ubiquitination governs protein degradation, trafficking, signal transduction, and functional transitions. It plays a critical role in maintaining cellular homeostasis and mediating responses to endogenous and exogenous stressors [71]. Dysregulated ubiquitination is closely associated with diverse pathological processes, including neuroinflammation, cell death, impaired axonal regeneration, and immune dysfunction, and has emerged as a promising therapeutic target [72]. Furthermore, metabolic crosstalk between the nervous system and peripheral tissues plays a pivotal role in axonal regeneration. This crosstalk is driven by glucose metabolism, lipid metabolism, proteolytic degradation, mitochondrial bioenergetics, and systemic humoral factors. These findings suggest that establishing a systemically pro‐regenerative metabolic environment is critical for successful neural repair [73, 74, 75, 76].

The included studies employed diverse intervention strategies, including specific inhibitors, TCMFG, small‐molecule compounds, siRNAs, drugs, metabolites, plasmid‐based approaches, and metal ions. These approaches confer neuroprotection by modulating inflammatory pathways, oxidative stress, BBB integrity, glial scarring, and neurogenesis [77, 78, 79, 80, 81]. Representative agents include methylprednisolone [82], NeuroSTAT [83], amantadine [84], tranexamic acid, metabolic reprogramming, and nanoparticle‐based delivery systems [85]. Approximately 98% of small‐molecule drugs cannot cross the BBB, limiting CNS pharmacotherapy [86]. Structural modifications enhancing passive diffusion, reducing efflux recognition, or exploiting carrier‐mediated transport improve permeability [87]. Delivery systems include microneedles [88], nanoparticles [85], hydrogels [89], and composite carriers [90].

Our findings indicate that microglial‐neuronal crosstalk is integral to the pathophysiology of TBI and SCI. Microglia and neurons engage in dynamic bidirectional communication mediated by ion channels, receptors, and transporters. This interaction is central to synaptic homeostasis, neuroinflammatory regulation, and tissue remodeling, but exhibits a dual neuroprotective and neurotoxic nature [91, 92]. The underlying mechanisms encompass neuroprotective and reparative processes [20], neurotoxicity and inflammatory damage [16], signaling pathways, and epigenetic regulation [93].

Disease‐gene association analysis revealed that ischemia was the most significantly enriched pathological factor. Ischemia–reperfusion injury (IRI) is a secondary injury mechanism triggered by the restoration of blood flow following CNS ischemia. It manifests as a sequential “double‐hit” characterized by an initial ischemic insult followed by reperfusion‐mediated damage [94, 95]. The core mechanisms of IRI include post‐reperfusion oxidative stress bursts (excessive ROS generation), calcium overload, excitotoxicity, immune inflammatory dysregulation, BBB disruption, and mitochondrial dysfunction [96, 97, 98]. Mitochondria are the principal organelles governing cellular bioenergetics and apoptotic signaling. They serve as both initiators and central mediators of IRI pathophysiology. Key mitochondrial alterations include dysregulated mitochondrial dynamics (aberrant fusion and fission), impaired quality control, altered membrane potential, and reverse electron transport [99, 100].

## 5 Limitations

First, current evidence for PANoptosis after TBI and SCI is derived solely from rodent studies with heterogeneous model parameters and detection timing, limiting comparability across studies and lacking validation in large animal and human tissues. Second, some studies only detected downstream PANoptosis executioners without validating PANoptosome sensors/adaptors; simultaneous apoptosis, pyroptosis, and necroptosis markers do not confirm PANoptosome‐driven PANoptosis. Third, only two studies investigated microglia–neuron interactions, and none examined astrocytes based on the inclusion and exclusion criteria.

## 6 Future Prospects and Clinical Translation

Several topics are prominent in PANoptosis research related to TBI and SCI. These include mitochondrial oxidative stress [101], gut‐brain axis regulation [41], cell type‐specific immunity and inflammation [45], and neuroimmune microenvironment modulation [102]. Emerging evidence highlights that voltage‐dependent anion channel 1 (VDAC1) oligomerization drives mitochondrial dysfunction and mitochondrial ROS (mtROS) production, promoting PANoptosome assembly and PANoptosis in retinal IRI. Inhibiting VDAC1 oligomerization or scavenging mtROS suppresses PANoptosis [103]. This mitochondrial oxidative stress–PANoptosis axis extends to neurological diseases, where targeting VDAC1, mtROS, and PANoptosome components represents a promising therapeutic strategy for neuroprotection [101]. Future research should prioritize the combined detection of PANoptosome sensors, adaptors, and executioners to more accurately capture the onset and progression of PANoptosis. Animal model parameters should be standardized to simulate varying injury severity and better capture the impact of disease severity on PANoptosis. The temporal dynamics of PANoptosis also require further attention. Further studies should examine astrocyte functions and interactions among different CNS cells in TBI and SCI. Key molecules involved in PANoptosis may serve as prognostic biomarkers and therapeutic targets. New technologies, such as physical assistance [104], nanomaterials [105], and bio‐mediated approaches [106], could improve the stability and targeting of drug delivery across the BBB. These advances would facilitate the translation of PANoptosis‐related findings from basic research to clinical applications.

## 7 Conclusion

Following the initial insult in TBI and SCI, microglia and neurons become activated, ultimately inducing PANoptosis through various regulatory mechanisms. Targeted interventions against PANoptosomes can effectively preserve the immuno‐inflammatory balance, thereby alleviating secondary injury following TBI and SCI. Future research should prioritize the comprehensive detection of PANoptosomes, standardization of animal model parameters, investigation of CNS cell interactions, and the development of novel drug delivery methods. The IRI associated with these conditions also warrants dedicated investigation.

## Author Contributions

**Zhusheng Feng:** writing – original draft, resources, data curation, visualization, and formal analysis. **Yingnan Fan:** writing – original draft, resources, data curation, visualization, validation, and formal analysis. **Tianshu Du:** writing – original draft, visualization, formal analysis, and funding acquisition; **Peipei Huang:** writing – review and editing, supervision, and validation; **Yuqin Ye:** writing – review and editing, supervision, validation, and visualization. **Junjie Li:** writing – review and editing, supervision, validation, and funding acquisition.

## Funding

This work was financially supported by grants from the National Natural Science Foundation of China (No. 82572467) and Shaanxi Provincial Natural Science Basic Research Program (No. 2026JC‐YBMS‐0994).

## Ethics Statement

The authors have nothing to report.

## Conflicts of Interest

The authors declare no conflicts of interest.

## Supporting information


**Table S1:** PRISMA 2020 for abstracts checklist.
**Table S2:** Search strategy in PubMed.
**Table S3:** Search strategy in Web of Science.
**Table S4:** Search strategy in Corchrane Library.
**Table S5:** Research data on the included experiments.
**Figure S1:** Flow diagram of the literature screening. A total of 62 records were retrieved from the three databases (PubMed: 30; Web of Science: 32; Cochrane Library: 0). After removing duplicates, 37 unique records were analyzed. Nine non‐research articles were excluded, leaving 28 records for eligibility screening. Of these, 15 were excluded because they were unrelated to TBI/SCI or PANoptosis or relied solely on genomic, transcriptomic, proteomic, or metabolomic analyses without experimental validation. Thirteen articles underwent full‐text assessments. Following the full‐text review, three additional studies were excluded due to a lack of information on regulatory mechanisms or intervention strategies targeting PANoptosis or the absence of protein‐level markers for PANoptosis. Ultimately, 10 studies met the inclusion criteria and were included in the qualitative synthesis. SCI, spinal cord injury; TBI, traumatic brain injury.
**Figure S2:** Evaluation of animal experiments using CRIME‐Q. (A) Reporting quality. Items 1X, 6X, 8X, and 10X were rated as “yes” in all 10 studies; Items 2X and 5X were rated as “partly” in all 10 studies; Item 3X was rated as “yes” in 8 studies and “partly” in 2; Item 7X was rated as “yes” in 1 study, “partly” in 6, and “no” in 3; Item 9X was rated as “yes” in 4 studies, “partly” in 5, and “no” in 1 study; (B) Methodological quality. Items 2Y, 3Y, and 5Y were rated as “yes” in all 10 studies; Item 4Y was rated as “no” in 9 studies and “partly” in 1; (C) Risk of bias. Items 3Z, 5Z (1), 5Z (2), 5Z (3), 7Z (2) and 7Z (3) were rated as “unclear” in all 10 studies; Item 7Z (1) was rated as “unclear” in 7 studies and “no” in 3; Items 8Z (1), 8Z (2), and 10Z were rated as “yes” in all 10 studies. 1X: peer review; 2X: bench‐top/laboratory work related to establishing model‐reporting; 2Y: bench‐top/laboratory work related to establishing model‐methodology (technical quality); 3X: animals‐reporting; 3Y: animals‐methodology (technical quality); 3Z: selection bias (baseline characteristics); 4Y: sample size calculation; 5X: in vivo design and performance‐reporting; 5Y: in vivo design and performance‐methodology(technical); 5Z (1): selection bias (sequence generation); 5Z (2): performance bias (random housing); 5Z (3): detection bias (random outcome assessment); 6X: compliance with animal welfare regulations; 7X: blinding; 7Z (1): performance bias (blinding); 7Z (2): allocation bias (allocation concealment); 7Z (3): detection bias (blinding); 8X: congruency between methods and results; 8Z (1): attrition bias (incomplete outcome data); 8Z (2): reporting bias (selective outcome reporting); 9X: presentation of limitations; 10X: statement of potential conflict of interest; 10Z: publication bias (influence); NA: not applicable; X: quality of reporting; Y: methodological quality; Z: risk of bias; CRIME‐Q: critical appraisal of methodological (technical) quality, quality of reporting and risk of bias in animal research.
**Figure S3:** Crosstalk between microglia and neurons. AKT, protein kinase B; EVO, evodiamine; IL‐6, interleukin‐6; MCP‐1, monocyte chemoattractant protein‐1; NLRP12, NOD‐like receptors family pyrin domain containing 12; PI3K, phosphatidylinositol 3‐kinase; SCI, spinal cord injury; SIRPα, signal regulatory protein alpha; SKY, spleen tyrosine kinase; TBI, traumatic brain injury; TNF‐α, tumor necrosis factor‐α; ZBP1, Z‐DNA binding protein 1.
**Figure S4:** Analysis of common genes involved in PANoptosis in TBI and SCI. (A) GeneCards analysis identified 211 genes associated with TBI, 99 with SCI, and 174 with PANoptosis, with 18 genes common to all three categories; (B) Eighteen shared genes (CASP3, HIF1A, HMGB1, IL1B, MAPK3, MTOR, PTGS2, TNF, NFKB1, HMOX1, NFE2L2, SIRT1, TLR4, CASP1, PARP1, MMP2, NLRP3, and MAPK1) were interconnected within a unified network; (C) KEGG pathways enrichment revealed that the pertussis signaling pathway was the most significantly enriched; (D) Reactome pathways enrichment identified interleukin signaling as the most significantly enriched pathway; (E) Biological process (Gene Ontology) enrichment highlighted cellular response to chemical stimuli, cellular response to stress, and response to oxygen‐containing compounds as the top terms; (F) Molecular function (Gene Ontology) enrichment emphasized transcription factor binding, enzyme binding, identical protein binding, and protein binding; (G) Cellular component (Gene Ontology) enrichment indicated that caveola and membrane rafts were the most significant components; (H) Disease‐gene associations (DISEASES) enrichment identified ischemia as the most significantly associated disease; SCI, spinal cord injury; TBI, traumatic brain injury.

## Data Availability

Data sharing not applicable to this article as no datasets were generated or analysed during the current study.
