# Supplementary material for: PANoptosis In Microglia and Neurons Following Traumatic CNS Injury: A Systematic Review of Pathological Features and Therapeutic Implications
Source: CNS Neurosci Ther. 2026 Oct 13;32(10):e71208. doi: 10.1002/cns.71208 (PMC13659638; doi:10.1002/cns.71208)
Supplement: Supplementary file 1 [file CNS-32-e71208-s001.docx]

**TABLE S1 |** PRISMA 2020 for abstracts checklist.

| **Section and Topic** | **Item #** | **Checklist item** | **Reported (Yes/No)** |
| --- | --- | --- | --- |
| **TITLE** | | |  |
| Title | 1 | Identify the report as a systematic review. | Yes |
| **BACKGROUND** | | |  |
| Objectives | 2 | Provide an explicit statement of the main objective(s) or question(s) the review addresses. | Yes |
| **METHODS** | | |  |
| Eligibility criteria | 3 | Specify the inclusion and exclusion criteria for the review. | Yes |
| Information sources | 4 | Specify the information sources (e.g. databases, registers) used to identify studies and the date when each was last searched. | Yes |
| Risk of bias | 5 | Specify the methods used to assess risk of bias in the included studies. | Yes |
| Synthesis of results | 6 | Specify the methods used to present and synthesise results. | Yes |
| **RESULTS** | | |  |
| Included studies | 7 | Give the total number of included studies and participants and summarise relevant characteristics of studies. | Yes |
| Synthesis of results | 8 | Present results for main outcomes, preferably indicating the number of included studies and participants for each. If meta-analysis was done, report the summary estimate and confidence/credible interval. If comparing groups, indicate the direction of the effect (i.e. which group is favoured). | Yes |
| **DISCUSSION** | | |  |
| Limitations of evidence | 9 | Provide a brief summary of the limitations of the evidence included in the review (e.g. study risk of bias, inconsistency and imprecision). | Yes |
| Interpretation | 10 | Provide a general interpretation of the results and important implications. | Yes |
| **OTHER** | | |  |
| Funding | 11 | Specify the primary source of funding for the review. | Yes |
| Registration | 12 | Provide the register name and registration number. | Yes |

*From:*  Page MJ, McKenzie JE, Bossuyt PM, Boutron I, Hoffmann TC, Mulrow CD, et al. The PRISMA 2020 statement: an updated guideline for reporting systematic reviews. BMJ 2021;372:n71. doi: 10.1136/bmj.n71. This work is licensed under CC BY 4.0. To view a copy of this license, visit <https://creativecommons.org/licenses/by/4.0/>

| **TABLE S2** \| Search strategy in PubMed. | |
| --- | --- |
| **Search** | **Query** |
| #1 | Brain Injuries, Traumatic[Mesh] |
| #2 | Traumatic Brain Injury |
| #3 | Brain Trauma |
| #4 | Spinal Cord Injuries[MeSH] |
| #5 | Spinal Cord Injury |
| #6 | Spinal Cord Trauma |
| #7 | PANoptosis |
| #8 | PANoptosome |
| #9 | PANoptotic |
| #10 | ((Brain Injuries, Traumatic[Mesh]) OR (Traumatic Brain Injury)) OR (Brain Trauma) |
| #11 | ((Spinal Cord Injuries[MeSH]) OR (Spinal Cord Injury)) OR (Spinal Cord Trauma) |
| #12 | ((PANoptosis) OR (PANoptosome)) OR (PANoptotic) |
| #13 | (((Brain Injuries, Traumatic[Mesh]) OR (Traumatic Brain Injury)) OR (Brain Trauma)) OR (((Spinal Cord Injuries[MeSH]) OR (Spinal Cord Injury)) OR (Spinal Cord Trauma)) |
| #14 | ((((Brain Injuries, Traumatic[Mesh]) OR (Traumatic Brain Injury)) OR (Brain Trauma)) OR (((Spinal Cord Injuries[MeSH]) OR (Spinal Cord Injury)) OR (Spinal Cord Trauma))) AND (((PANoptosis) OR (PANoptosome)) OR (PANoptotic)) |

| **TABLE S3 \|** Search strategy in Web of Science. | |
| --- | --- |
| **#** | **Search Query** |
| 1 | TS=(Brain Injuries, Traumatic) |
| 2 | ALL=(Brain Trauma) |
| 3 | ALL=(Traumatic Brain Injury) |
| 4 | TS=(Spinal Cord Injuries) |
| 5 | ALL=(Spinal Cord Injury) |
| 6 | ALL=(Spinal Cord Trauma) |
| 7 | ALL=(PANoptosis) |
| 8 | ALL=(PANoptosome) |
| 9 | ALL=(PANoptotic) |
| 10 | #1 OR #2 OR #3 |
| 11 | #4 OR #5 OR #6 |
| 12 | #7 OR #8 OR #9 |
| 13 | #10 OR #11 |
| 14 | #12 AND #13 |

| **TABLE S4 \|** Search strategy in Corchrane Library. | |
| --- | --- |
| **No.** | **Query** |
| #1 | MeSH descriptor: [Brain Injuries, Traumatic] explode all trees |
| #2 | Traumatic Brain Injury |
| #3 | Brain Trauma |
| #4 | MeSH descriptor: [Spinal Cord Injuries] explode all trees |
| #5 | Spinal Cord Injury |
| #6 | Spinal Cord Trauma |
| #7 | PANoptosis |
| #8 | PANoptosome |
| #9 | PANoptotic |
| #10 | #1 OR #2 OR #3 |
| #11 | #4 OR #5 OR #6 |
| #12 | #7 OR #8 OR #9 |
| #13 | #10 OR #11 |
| #14 | #12 AND #13 |

| **TABLE S5** \| Research data on the included experiments. | | | | | | | |
| --- | --- | --- | --- | --- | --- | --- | --- |
| **Disease** | **Species** | **Model** | **Injury site** | **Specific parameter** | **Cell marker** | **PANoptosis detection** | **Reference** |
| SCI | SD rats | CCI | T10 spinal cord | The striker tip diameter, velocity, depth, and duration were 2.0 mm, 0.5 m/s, 1.0 mm, and 1 s, respectively. | Iba1 | Day 3, Day 7 | Xu 2026 [40] |
| TBI | C57BL/6 mice | CCI | Cortex under right parietal bone | The velocity, depth, and duration were 3.0 m/s, 1.0 mm, and 0.1 s, respectively. | NeuN | Day 14 | Xie 2026 [41] |
| TBI | C57BL/6 mice | CCI | Left cortex | The striker tip diameter, velocity, depth, and duration were 3.0 mm, 6.0 m/s, 0.6 mm, and 0.1 s, respectively. | Iba1 | Day 3 | Shuang 2026 [42] |
| SCI | Wistar rats | Contusion | T10 spinal cord | The impactor rod weighed 10.0 g and had a height of 25.0 mm. | Iba1 | Day 7 | Liu 2026 [43] |
| SCI | C57BL/6 mice | Contusion | T10 spinal cord | The tip diameter was 1.2 mm, weight was 5.0 g, and height was 30 mm. | NeuN | After SCI | Jiang 2026 [44] |
| TBI | C57BL/6 mice | CCI | Cortex under anterior fontanelle and the sagittal suture | Low pressure: 100.0 kPa; high pressure: 200.0 kPa; depth: 1 mm. | NeuN | Day 1 | Chen 2026 [45] |
| TBI | C57BL/6 mice | CCI | Cortex under right parietal bone | The impactor tip diameter, velocity, and depth were 2.5 mm, 3.5 m/s, and 3.0 mm, respectively. | Iba1 | Day 1 | Zheng 2025 [46] |
| TBI | C57BL/6 mice | CCI | Cortex under left parietal bone | The speed, depth, and duration were 6.0 m/s, 1.4 mm, and 0.05 s, respectively. | NeuN | Day 21 | Xu 2025 [47] |
| SCI | C57BL/6 mice | Contusion | Thoracic spinal cord | NS | NeuN | Day 3 | Lou 2024 [48] |
| SCI | C57BL/6 mice | Contusion | T9/T10 spinal cord | Impactor force of 50.0 kdyn | NR | Day 3 | Bai 2024 [49] |
| CCI: controlled cortical impact; Iba1: ionized calcium-binding adapter molecule 1; kdyn: kilodyne; NeuN: neuronal nuclei; NR: not reported; NS: not specified; SD: Sprague-Dawley; SCI: spinal cord injury; TBI: traumatic brain injury. | | | | | | | |

**FIGURE S1** | Flow diagram of the literature screening. A total of 62 records were retrieved from the three databases (PubMed: 30; Web of Science: 32; Cochrane Library: 0). After removing duplicates, 37 unique records were analyzed. Nine non-research articles were excluded, leaving 28 records for eligibility screening. Of these, 15 were excluded because they were unrelated to TBI/SCI or PANoptosis or relied solely on genomic, transcriptomic, proteomic, or metabolomic analyses without experimental validation. Thirteen articles underwent full-text assessments. Following the full-text review, three additional studies were excluded due to a lack of information on regulatory mechanisms or intervention strategies targeting PANoptosis or the absence of protein-level markers for PANoptosis. Ultimately, 10 studies met the inclusion criteria and were included in the qualitative synthesis. SCI: spinal cord injury; TBI: traumatic brain injury.

**FIGURE S2 |** Evaluation of animal experiments using CRIME-Q. **(A) Reporting quality.** Items 1X, 6X, 8X, and 10X were rated as "yes" in all 10 studies; items 2X and 5X were rated as "partly" in all 10 studies; item 3X was rated as "yes" in 8 studies and "partly" in 2; item 7X was rated as "yes" in 1 study, "partly" in 6, and "no" in 3; item 9X was rated as "yes" in 4 studies, "partly" in 5, and "no" in 1 study; **(B) Methodological quality.** Items 2Y, 3Y, and 5Y were rated as "yes" in all 10 studies; item 4Y was rated as "no" in 9 studies and "partly" in 1; **(C) Risk of bias.** Items 3Z, 5Z(1), 5Z(2), 5Z(3), 7Z(2) and 7Z(3) were rated as "unclear" in all 10 studies; item 7Z(1) was rated as "unclear" in 7 studies and "no" in 3; items 8Z(1), 8Z(2), and 10Z were rated as "yes" in all 10 studies. 1X: peer review; 2X: bench-top/laboratory work related to establishing model-reporting; 2Y: bench-top/laboratory work related to establishing model-methodology (technical quality); 3X: animals-reporting; 3Y: animals-methodology (technical quality); 3Z: selection bias (baseline characteristics); 4Y: sample size calculation; 5X: in vivo design and performance-reporting; 5Y: in vivo design and performance-methodology(technical); 5Z (1): selection bias (sequence generation); 5Z (2): performance bias (random housing); 5Z (3): detection bias (random outcome assessment); 6X: compliance with animal welfare regulations; 7X: blinding; 7Z (1): performance bias (blinding); 7Z (2): allocation bias (allocation concealment); 7Z (3): detection bias (blinding); 8X: congruency between methods and results; 8Z (1): attrition bias (incomplete outcome data); 8Z (2): reporting bias (selective outcome reporting); 9X: presentation of limitations; 10X: statement of potential conflict of interest; 10Z: publication bias (influence); NA: not applicable; X: quality of reporting; Y: methodological quality; Z: risk of bias; CRIME-Q: critical appraisal of methodological (technical) quality, quality of reporting and risk of bias in animal research.

**FIGURE S3** | Crosstalk between microglia and neurons. AKT: protein kinase B; EVO: evodiamine; IL-6: interleukin-6; MCP-1: monocyte chemoattractant protein-1; NLRP12: NOD-like receptors family pyrin domain containing 12; PI3K: phosphatidylinositol 3-kinase; SCI: spinal cord injury; SIRPα: signal regulatory protein alpha; SKY: spleen tyrosine kinase; TBI: traumatic brain injury; TNF-α: tumor necrosis factor-α; ZBP1: Z-DNA binding protein 1.

**FIGURE S4 |** Analysis of common genes involved in PANoptosis in TBI and SCI. (A) GeneCards analysis identified 211 genes associated with TBI, 99 with SCI, and 174 with PANoptosis, with 18 genes common to all three categories; (B) Eighteen shared genes (CASP3, HIF1A, HMGB1, IL1B, MAPK3, MTOR, PTGS2, TNF, NFKB1, HMOX1, NFE2L2, SIRT1, TLR4, CASP1, PARP1, MMP2, NLRP3, and MAPK1) were interconnected within a unified network; (C) KEGG pathways enrichment revealed that the pertussis signaling pathway was the most significantly enriched; (D) Reactome pathways enrichment identified interleukin signaling as the most significantly enriched pathway; (E) Biological process (Gene Ontology) enrichment highlighted cellular response to chemical stimuli, cellular response to stress, and response to oxygen-containing compounds as the top terms; (F) Molecular function (Gene Ontology) enrichment emphasized transcription factor binding, enzyme binding, identical protein binding, and protein binding; (G) Cellular component (Gene Ontology) enrichment indicated that caveola and membrane rafts were the most significant components; (H) Disease-gene associations (DISEASES) enrichment identified ischemia as the most significantly associated disease; SCI: spinal cord injury; TBI: traumatic brain injury.
